# Supplementary material for: Positive Allosteric Modulators of SERCA Pump Restore Dendritic Spines and Rescue Long-Term Potentiation Defects in Alzheimer’s Disease Mouse Model
Source: Int J Mol Sci. 2023 Sep 12;24(18):13973. doi: 10.3390/ijms241813973 (PMC10530588; doi:10.3390/ijms241813973)
Supplement: Supplementary file 1 [file ijms-24-13973-s001.zip › ijms-2596651-supplementary.pdf]

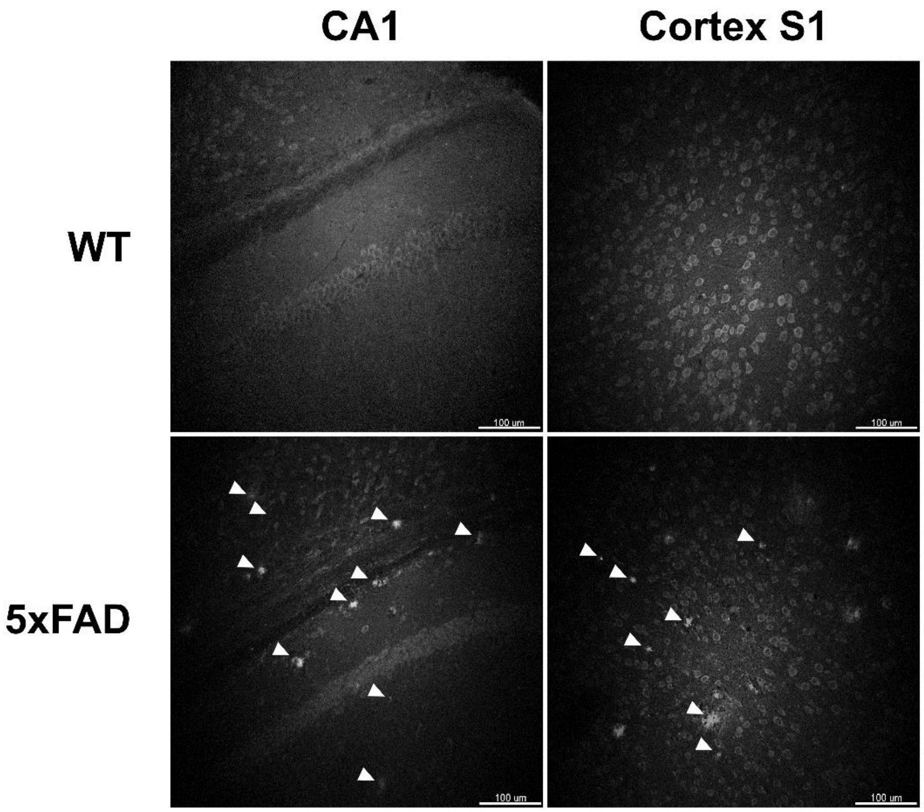

Fig S1: Representative images of amyloid plaques (indicated by white triangles) in the brains of 6-month-old 5xFAD and WT mice in the hippocampal CA1 region and the cortical S1 region. The slice thickness was 100 µm, and images were captured at 20x magnification. The length of the scale bar segment is 100 µm. For staining, an indirect immunohistochemical method was employed using primary antibodies against Aβ-peptide (6E10, 1:500, Biolegend, #SIG-39320) and secondary antibodies Alexa Fluor-488 (1:1000, ThermoFisher Scientific, #A-11001).
